# Epidural Abscess: A Cause of Back Pain That Must Not Be Missed

**DOI:** 10.7759/cureus.14376

**Published:** 2021-04-09

**Authors:** Inês Egídio de Sousa, Margarida Brito Monteiro, Maria Piteira, António Cuco, Paulo Telles Freitas

**Affiliations:** 1 Internal Medicine Department, Hospital São Francisco Xavier, Lisbon, PRT; 2 Internal Medicine Department, Hospital Prof. Doutor Fernando Fonseca, Amadora, PRT; 3 Internal Medicine Department, Hospital do Espirito Santo de Évora, Évora, PRT; 4 Neurosurgery Department, Hospital Egas Moniz, Lisbon, PRT; 5 Intensive Care Unit, Hospital Prof. Doutor Fernando Fonseca, Amadora, PRT

**Keywords:** back pain, cervical pain, tetraparesis, epidural abscess, spinal cord compression, spinal pain

## Abstract

Low back and cervical pain are common emergency department complaints. Most cases represent low-risk pathologies but in rare cases, it can be a symptom of a serious underlying disease and the distinction can be challenging. A case is reported of a 58-year-old male with recurrent cervical pain requiring several emergency department visits. He eventually presented with intense cervical and low back pain, tetraparesis, and dyspnea. His condition deteriorated in less than 24 hours, evolving with fever, shock, and respiratory insufficiency and was admitted to the Intensive Care Unit for aminergic support, invasive mechanical ventilation and started on empiric antibiotics. Investigations revealed raised inflammatory markers and a cervical pre-vertebral purulent collection associated with an epidural collection at the level of C2 to C5 with spinal cord compression. The patient underwent transoral drainage and removal of the posterior C1 arch along with C2-C3-C4-C5 laminectomy. Staphylococcus aureus was isolated in the blood, pus, and bone. Re-intervention was required due to persistent pre-vertebral abscesses with clinical improvement thereafter. Eight weeks of antibiotic treatment were completed in total. The patient was discharged after 72 days of hospitalization with significant improvement in force and autonomy. Epidural abscess is a rare finding that can cause rapid irreversible neurological deterioration. Clinical suspicion must be high for an early diagnosis and treatment, essential for a better prognosis.

## Introduction

Spinal pain is highly prevalent in first-world countries and is a common cause for emergency department visits [1]. Most times it represents self-limited and low-risk conditions, where imaging exams are inappropriate and represent unnecessary radiation exposure and increased healthcare costs. However, in rare cases, acute spinal pain can be caused by serious underlying diseases and the identification of these cases is challenging and demanding. A comprehensive evaluation looking for red-flag signs and symptoms remains a cornerstone on the evaluation of acute spinal pain [2]. We present a case of a very rare pathology as the cause of a very common presentation in the emergency setting.

## Case presentation

We report a case of a 58-year-old male with a background of hypertension and gonarthrosis who presented several times in the previous month to the emergency department with recurrent cervical pain.

He was admitted with intense cervical and low back pain, 22 days after the first hospital visit, and a four-day history of reduced muscle strength in the upper limbs and a new onset of dyspnea.

On examination, the patient was found to have tachycardia and tachypnea with unremarkable chest auscultation. A swelling of the posterior wall of the oro and hypopharynx was evident on the examination. Neurological exam revealed reduced strength on head flexion and rotation as well as shoulder elevation, hypotonic tetraparesis (strength 1/5 in the proximal upper limb muscles and 4/5 in the remaining upper and lower limbs), absent radial reflexes and reduced ankle reflexes. His condition deteriorated rapidly in less than 24 hours developing fever and shock with multiorgan failure. He was admitted to the intensive care unit requiring mechanical ventilation plus aminergic support and started on empiric antibiotics (meropenem, ampicillin, and acyclovir).

**Figure 1:**
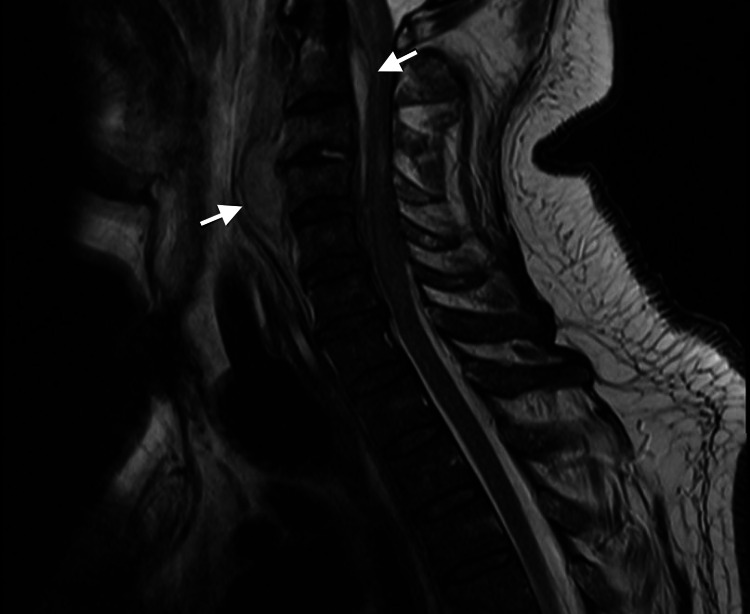
Sagittal T2 MRI revealing an anterior epidural abscess (C1-C4) compressing the spinal cord with high T2 signal and a prevertebral abscess (C3-C4).

**Figure 2:**
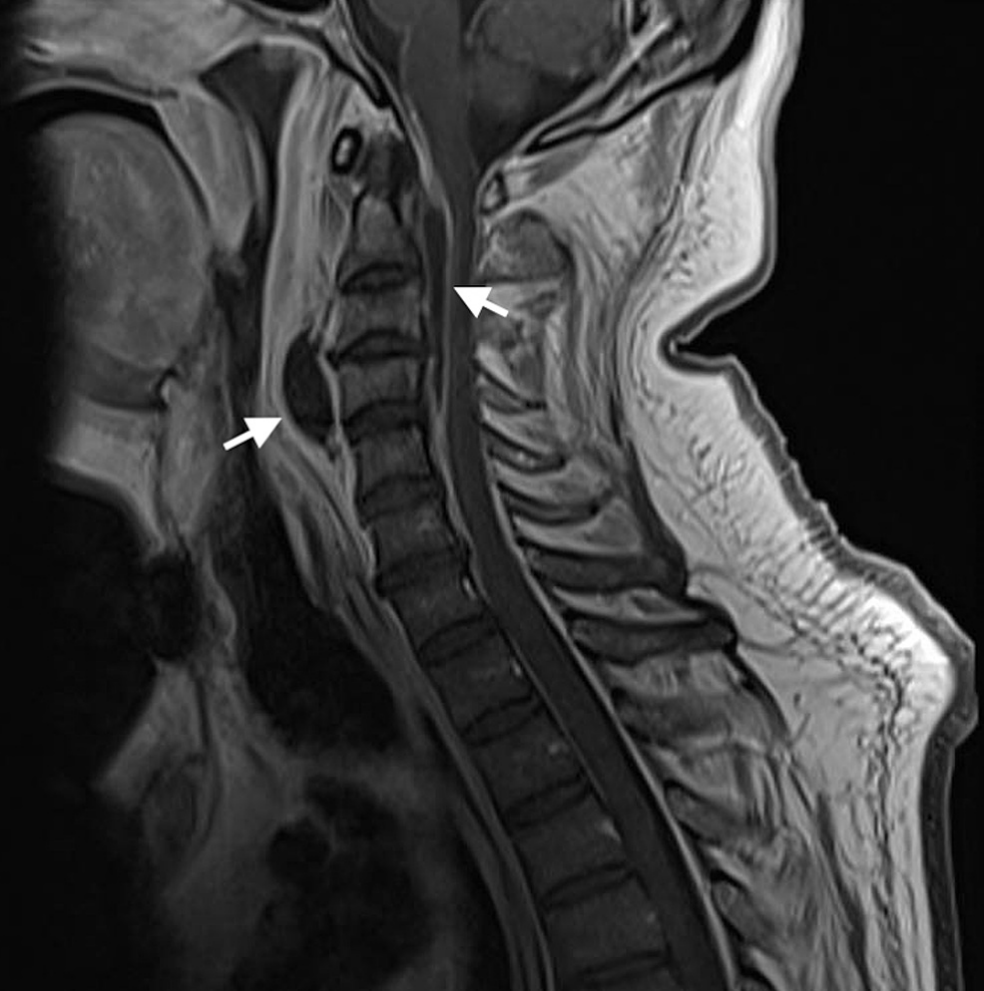
Sagittal T1 MRI revealing hypointense epidural and prevertebral abscesses.

From the investigations performed, the following are of remark: raised inflammatory markers (c-reactive protein [CRP] 33 mg/dL); raised protein, low glucose levels (with normal serum glucose), and 216 cells (predominantly mononuclear) in the cerebrospinal fluid; normal echocardiogram and a cervical spine magnetic resonance imaging (MRI) revealing a cervical pre-vertebral collection associated with an epidural collection at the level of C2 to C5 with spinal cord compression (Figures 1, 2).

The diagnosis of pre-vertebral and epidural abscesses was thus established as the cause for spinal cord compression and septic shock. The patient underwent transoral abscess drainage by otorhinolaryngology (ENT) and decompression of the spinal cord and drainage of the epidural collection by removal of the posterior C1 arch along with C2-C3-C4-C5 laminectomy by neurosurgery. The antibiotic therapy was switched to flucloxacillin after a positive oxacillin sensitive Staphylococcus aureus in the blood, pus, and bone, with clindamycin being added five days later due to persistent fever and high inflammatory parameters.

A computed tomography (CT) scan on day 9 postoperative revealed a persistent small pre-vertebral abscess. The patient was taken for further transoral drainage and a tracheostomy performed by ENT. Following the procedure, there was marked clinical improvement with the weaning of aminergic and ventilatory support. There was progressive improvement of neurological deficits under rehabilitation and effective pain control achieved with transdermal fentanyl 50 mcg and pregabalin 300 mg/day. A repeat CT two months after the first intervention ruled out complications or signs of spinal instability. Eight weeks of targeted antibiotics were completed. After 72 days of hospitalization he was discharged to a rehabilitation unit with improved muscle strength (grade 2/5 in the right proximal upper limb and 4/5 in the remaining muscles), autonomous standing, and feeding.

## Discussion

Spinal epidural abscess (SEA) is a localized infection situated between the thecal sac of the spinal cord and the spinal ligaments and vertebrae. It is a rare entity, with an incidence of 0.2-2 cases per 10,000 hospital admissions. Cervical SEA accounts for only 19% of all SEAs and is a more urgent condition as the smaller epidural space is less permissive to inflammation and due to the fragility of the atlantoaxial joint. Also, spinal cord compression can impact breathing due to diminished diaphragmatic innervation from C3, C4, and C5 [3,4].

The vague presentation of this the disease makes the diagnosis very challenging. Prolonged delays in the diagnosis are common and patients are often examined by multiple physicians before a diagnosis is made [5]. In this case, the fact that the patient presented pain at rest, without improvement with conservative care and motivating several visits to the emergency department for continued complaint should have raised concern for serious pathology and lead to earlier investigation. Only after the development of major red flags, such as neurologic compromise and fever, imaging exams were requested and diagnosis of SEA was reached [2].

Back pain, fever, and neurological deficit are the classic triad of symptoms of SEA, although few patients show all three at presentation, like the case presented. Pain characteristics of cervical epidural abscesses are commonly described as localized cervical pain or stiffness but can also present as nonspecific or irradiated pain to head or low back. Airway inspection and a full neurologic examination including cranial nerves is mandatory and may elicit sensorimotor deficit; however, a normal neurologic examination does not exclude the diagnosis [4].

Identification of the risk factors may help in the early establishment of the diagnosis. The most common risk factors are: diabetes, immunodeficiency, obesity, trauma, anesthetic procedures as spinal anesthesia and epidural catheter placement, intravenous drug abuse, and surgical instrumentation [4]. This patient did not present any known risk factor, making the diagnosis less evident.

Regarding the route of infection, bacteria reach the epidural space by hematogenous spread, contiguous spread (e.g., vertebral osteomyelitis), or direct inoculation (e.g., recent spinal procedure). However, in a significant number of patients there is no clear reported origin of infection for the epidural abscess [4,6]. In this case, the epidural space was probably reached by contiguous spread from the prevertebral abscess. The source of the prevertebral disease was not clearly identified but could have been secondary to an underdiagnosed upper respiratory tract infection or local inflammatory disease.

MRI remains the modality of choice with the greatest diagnostic accuracy and it established the diagnosis in this patient. The reported predictive values include sensitivity up to 95% and specificity over 90% [4]. As part of the evaluation, inflammatory markers should be ordered; although these markers are not specific to SEA, they are supportive of a diagnosis. Pathogen identification is not always possible from the blood or cerebrospinal cultures [4]. Staphylococcus aureus is the most common pathogen for SEA and was isolated both in blood, pus and bone in our patient [3].

On the basis of the available evidence, surgical decompression and abscess evacuation followed by specific culture-guided intravenous antibiotics is the best course of action for these patients and was essential for the good outcome of our patient [4]. Treatment duration for SEAs has not been specifically studied in a randomized clinical trial; however, 4-8 weeks is generally advised [6]. Surgery should be performed as soon as possible especially in cervical abscesses, in patients developing neurologic signs or with worsening inflammatory markers [3].

## Conclusions

Cervical and low back pain mostly represents low-risk pathologies with no need for radiological investigation; however, it can rarely be a symptom of a serious underlying disease. Identifying a serious pathology causing spinal pain can be challenging particularly in the absence of red flags so clinical suspicion should be high. Neurological deficits should be sought and prompt rapid radiological investigation.

Cervical epidural abscess is a rare condition that can cause rapid irreversible neurological deterioration and have devastating consequences. Increased awareness of the disease is essential for rapid recognition as early treatment is vital to prevent irreversible neurological damage, having a tremendous impact on the prognosis.
